# Pathological Preoccupation with Healthy Eating (Orthorexia Nervosa) in a Spanish Sample with Vegetarian, Vegan, and Non-Vegetarian Dietary Patterns

**DOI:** 10.3390/nu12123907

**Published:** 2020-12-21

**Authors:** María Laura Parra-Fernández, Maria Manzaneque-Cañadillas, María Dolores Onieva-Zafra, Elia Fernández-Martínez, Juan José Fernández-Muñoz, María del Carmen Prado-Laguna, Anna Brytek-Matera

**Affiliations:** 1 Department of Nursing, Physiotherapy and Occupational Therapy, Faculty of Nursing of Ciudad Real, Universidad de Castilla-La-Mancha, 13001 Ciudad Real, Spain; marialaura.parra@uclm.es (M.L.P.-F.); maria.manzaneque@alu.uclm.es (M.M.-C.); mariadolores.onieva@uclm.es (M.D.O.-Z.); carmina.prado@uclm.es (M.d.C.P.-L.); 2 Department of Nursing, University of Huelva, 21004 Huelva, Spain; elia.fernandez@denf.uhu.es; 3 Department of Psychology, University Rey Juan Carlos, Alcorcón, 28933 Móstoles, Madrid, Spain; Juanjose.fernandez@urjc.es; 4 Institute of Psychology, University of Wroclaw, 50-137 Wroclaw, Poland

**Keywords:** orthorexia nervosa, vegetarians, vegans, ORTO-11-ES, FCQ-SP

## Abstract

Orthorexia nervosa (ON) has been defined as an obsessive and pathological attitude towards healthy nutrition. The aim of this study was to compare individuals who followed a vegan, vegetarian, and omnivore diet in terms of ON behaviors and to examine their prime motivations, attitudes, and behaviors towards food. The Spanish version of the ORTO-15 test — ORTO-11-ES — and the Food Choice Questionnaire (FCQ-SP) were used with a demographic questionnaire in an online survey disseminated among the social networks of different vegetarian associations and the general population. Of 466 individuals, 55% followed an omnivore diet, 23.5% were vegetarian and 21.7% were vegan. Results revealed relationships between type of diet and FCQ-SP dimensions for: health and natural content *(H* = 8.7, *p* < 0.05), sensory appeal (*H* = 11.4, *p* < 0.01), weight control (*H* = 40.4, *p* < 0.01), and familiarity (*H* = 37.3, *p* < 0.01). Our results confirm the findings of recent studies showing that individuals who follow a vegan or vegetarian diet are more likely to develop a pathological preoccupation with healthy eating versus omnivores. Further studies are required to determine the potential lines of action for the prevention of ON.

## 1. Introduction

An individual’s diet and lifestyle have important implications on many health-related aspects. The number of individuals who choose to adopt different types of diets is increasing; a trend that stems from several reasons, including a compassion towards animals, the desire to protect the environment, or to prevent or treat certain illnesses [1,2]. Vegetarian diets include an array of dietary patterns and, generally, there is no consumption of animal protein, specifically, red meat, poultry and/or fish. More specifically, lacto-ovo vegetarians include dairy and eggs in their diet, whereas vegans exclude all meat or animal derived products. Certain studies also include other categories of vegetarians, such as semi vegetarians or pescatarians, who include limited amounts of meat and/or fish in their diets [3]. It is estimated that 1.5% of the Spanish population follows a vegetarian diet [4]. The studies performed to date have highlighted the major importance of health and ethical aspects as factors which determine the choice behind following these type of diets [5]. When appropriately planned, these diets can provide many health benefits. The problem arises when they are taken to extremes, triggering eating disorders [6,7].

In 1997, the American physician, Steven Bratman [8] was the first to define Orthorexia Nervosa (ON), characterized by the appearance of a pathological obsession for healthy eating. This term comes from the Greek, which means *ὀρθóς* (correct) and *ὄρεξις* (appetite), thus referring to a correct or fair appetite [8]. The relationship between ON and eating disorders has long been studied, and there is strong empirical support for its association with eating behavior disorders [9]. Furthermore, some studies point to certain similarities with obsessive compulsive disorders [10,11]. The symptoms which accompany ON have been updated and refined over time [12,13,14]. People with ON experience anxiety and a tendency for negative emotions in uncertain situations, even with minimal potential threat [15]. Symptoms of ON include concern, obsessions, compulsions, rituals, and avoidance, which are used to reduce uncertainty and negative affect in order to perceive a sense of control over a person’s diet and surrounding environment [14,15,16].

Relatively recent studies have been performed to examine the possible relationship between ON and the acquirement of certain norms and restrictions regarding specific foods in an individual’s diet. Overall, previous studies examining the prevalence of ON among vegetarians and vegans report mixed results. Some researchers have found that vegetarians obtain higher scores on the ON measurements [17,18,19,20], whereas others do not report significant differences between groups [21,22]. One of the first research, conducted by Missbach et al. [17] among a sample of 1029 German-speaking people, found that vegetarian and vegan dietary patterns were associated with ON scores. Another study was conducted by Dell’Osso et al. [23] on a sample of 2130 students, finding that vegetarian or vegan subjects presented a significantly higher rate of orthorexic symptoms than individuals following an omnivorous diet. Heiss et al. [24] also examined ON in individuals with a restricted consumption of meat and found a significant link between vegetarian diet and ON. In contrast, a study by Cicekoglu and Tunçay [22] on a sample of 62 people, of whom 31 were vegan/vegetarian and 31 were non-vegan/non-vegetarian, did not find any association between ON and those individuals who decided to follow these types of diets. The variability of these data may be explained by several reasons, ranging from the lack of uniformity in the definition of certain specific vegetarian diets (e.g., vegans, lacto-ovo vegetarians, etc.) to the different scales used to measure ON [21,25]. This suggests the need for further research to resolve some of the uncertainties of this disorder regarding the possible relations between the avoidance of meat and ON.

ON has been studied extensively for the past two decades, which have an effect on an increased amount of ON measures. At present, ten ON tools have been developed (for more details, see review by Opitz et al. [26]). Four of them, Bratman Orthorexia Test [27], ORTO-15 [28], the Düsseldorfer Orthorexia Scale [29], and the Eating Habits Questionnaire [15] have been widely utilized to evaluate ON. The ORTO-15 is the most frequently used self-report measure within the field of ON research [14]. Some methodological problems related to the ORTO-15 have been identified [14,30]. Its two main limitations which might call into question the accuracy of the results are: measuring ON prevalence and the unstable factorial structure of the ORTO-15 across different populations [31]. Despite this, the ORTO-15 development was a milestone in the understanding of ON [31]. In addition, some researchers acknowledged the ORTO-15 being at present the only well accepted method of screening for ON symptoms (for more details, see review by Valente et al. [32]). It is worth pointing out that in the present study we used the ORTO-11-ES neither not for assessing the ON prevalence nor as a diagnostic measure but for investigating ON behaviors.

Acquiring a better understanding of the possible effects of eating behaviors among individuals with diets where the consumption of meat is restricted, is particularly important for two reasons. First, veganism has become more popular over the last 15 years, with a greater proportion of the Spanish population adopting veganism [4]. In second place, controversy exists among those who affirm that vegan diets have numerous health benefits, such as a reduction in the risk of cardiovascular disease, cancer, obesity, and type 2 diabetes [33,34,35,36]. The relative scarcity of information specifically centered on the possible relationship of ON among this population is therefore notable and it is this gap of knowledge which drives the present research.

No hypotheses were made due to the exploratory nature of the study. Therefore, the aim of this study was to compare individuals with a vegan, vegetarian, and omnivore diet in terms of ON behaviors, as well as examine the prime motivations, attitudes, and behaviors towards food among the different groups. This study attempted to overcome the weaknesses of previous research by recruiting a sample comprising large communities of vegan individuals and comparing these to demographically comparable omnivores in order to facilitate future research on ON.

## 2. Materials and Methods

### 2.1. Participants and Procedure

The recruitment of participants took place by reaching out to social networks among different vegetarian and other local associations. The questionnaires were administrated online, via a link attached to an email with an invitation that included a clear and comprehensive description of the study. All participants gave their permission to be part of the study, and they provided informed voluntary written consent prior to initiating the survey via an online consent form. They were informed that their participation was voluntary and anonymous. Participants were asked to complete the ORTO-11-ES, the Food Choice Questionnaire (FCQ-SP), sociodemographic variables and diet characteristics. The subjects did not receive any payment or any other incentives to complete the survey. Due to the exploratory nature of the study, no exclusion criteria were applied and all the individuals who agreed to participate were considered eligible. Ethical approval for this study was obtained from Ethics committee at General University Hospital of Ciudad Real (no. C-240). All procedures performed in our study were in accordance with the 1964 Helsinki declaration (adopted by the 18th World Medical Association General Assembly, Helsinki, Finland) and its later amendments or comparable ethical standards.

### 2.2. Measures

#### 2.2.1. Demographic Information

The sociodemographic forms gathered information on the age, sex, height, and weight of participants. The Body Mass Index (BMI) of each participant was calculated based on the self-reported height and weight (BMI = weight (kg)/(height (m))^2^). The information related to the participant’s diet was also self-reported by the participant, by stating whether they were vegetarians, vegans, or omnivores (a yes-no question).

#### 2.2.2. ORTO-11-ES

The ORTO-11-ES [37] is based on the original ORTO-15 questionnaire in Italian [28]. This scale is based on the original questionnaire by Bratman [8]. It is used to investigate obsessive behavior related to the selection and preparation of food, habits of food consumption and attitudes towards healthy food, and is complemented by characteristics of obsessive-compulsive disorder, as measured by the Minessota Multiphasic Personality Inventory [38]. This tool consists of 15 self-report multiple choice items using a 4-point Likert-type scale (always, often, sometimes, never) to measure three underlying factors related to eating behavior: cognitive rational, clinical, and emotional aspects. The Italian validation [28] showed a good prediction capacity for a cut-off value of 40, an effectiveness of 73.8%, a sensitivity of 55.6% and a specificity of 75.8%. The ORTO-11-ES [39] is a three-factor solution, with the inclusion of 11 items (items 1, 2, 3, 4, 6, 7, 9, 10, 11, 12, and 13) from the original ORTO-15 test, and four removed items (items 5, 8, 14, and 15). The internal consistency of this abbreviated, 11-item version (ORTO-11-ES) is acceptable with a Cronbach’s alpha of 0.80 [39]. In the ORTO-11-ES a score of 25 or lower indicates tendency to ON or a risk of developing ON, while a total score of more than 25 identifies the absence of ON. In addition, this questionnaire has demonstrated 79.5% effectiveness, 75% sensitivity, and specificity 79.6%) [38,39]. In the current sample, Cronbach’s alpha coefficient of the ORTO-11-ES was 0.760.

#### 2.2.3. Food Choice Questionnaire

The Food Choice Questionnaire (FCQ-SP) Spanish version is a self-report tool which consists of 34 items with multiple response options and employing a Likert scale with seven response possibilities, which range from “not at all important” to “very important”, to measure seven underlying factors related with the reasons for choosing food [40]. For this study, the authors applied the Spanish factorial solution composed by seven dimensions (34 items): mood, health and natural content, sensory appeal, weight control, convenience, familiarity, and price. The FCQ-SP had a Cronbach’s α coefficient of 0.90 [40]. In the current sample, Cronbach’s alpha coefficient of the FCQ-SP was 0.921.

### 2.3. Statistical Analysis

The analyses were performed with SPSS 25.0. The characteristics of the participants were determined by using frequency analysis and mean values. Moreover, the Chi-squared test (*p* < 0.05) was used to compare sex, marital status, and BMI, type of diet and ON. The Kolmogorov–Smirnov test (*n* > 50) was applied to determine whether ORTO-11-ES and subscales of the FCQ-SP had a normal distribution. If the scores obtained *p* < 0.05 the Mann–Whitney U test (for ON) and the Kruskal–Wallis H test (for type of diet) were applied in order to determine the significant differences. Finally, Spearman’s Rho correlations were used between ON and subscales of the FCQ-SP. Moreover, to reduce multiplicity problems, the α level was set at 0.05, with three comparisons.

## 3. Results

A total of 466 individuals agreed to participate in the survey, of these 54.9% followed an omnivore diet, 23.4% were vegetarian, and 21.7% were vegan. The mean age was 32.2 years (SD = 11.08) with a range from 18 to 73. The mean age for females was 31.2 (SD = 10.9) and for males it was 33.6 (SD = 12.0). For each type of diet (the age mean) was vegan (M = 33.3, SD = 11.1), vegetarian (M = 29.2, SD = 11.1), and omnivore (M = 33.0, SD = 11.7).

Table 1 shows the sociodemographic characteristics of individuals consuming the three types of diet. For sex, *p* = 0.09 indicating that there were no differences between groups in the percentage of women. For marital status, omnivores were relatively more likely to be married than vegans and vegetarians. Omnivores were more likely to be overweight than individuals in the other two categories and vegans had the highest risk of ON.

Table 2 shows the Kruskal–Wallis H test dimensions of the FCQ-SP and type of diet. The relationships between type of diet and dimensions of the FCQ-SP were significant for: health and natural content (*H* = 8.7, *p* < 0.05), sensory appeal (*H* = 11.4, *p* < 0.01), weight control (*H* = 40.3, *p* < 0.01), and familiarity (*H* = 37.2, *p* < 0.01). Finally, the remaining dimensions on the FCQ-SP (price, mood, and convenience) did not display significant relationships regarding type of diet (*p* > 0.05). The post hos analyses for the natural content showed just significance differences between vegans and omnivorous (*H* = −45.1, *p* < 0.01); for sensory appeal between vegetarian and omnivorous (*H* = 48.9, *p* < 0.01); weight control with significance differences between vegan and omnivorous (*H* = 94.4, *p* < 0.01) and vegetarian and omnivorous (*H* = 56.9, *p* < 0.01); and finally, post hoc analysis for familiarity shows significance differences between vegetarian and omnivorous (*H* = 87.5, *p* < 0.01) and vegan and omnivorous (*H* = −59.4, *p* < 0.01).

Table 3 shows the relationships between ON and dimensions of FCQ-SP; significant relationships were found for: mood (U = 21365.00, *p* < 0.01), health and natural content (U = 19107.50, *p* < 0.01), and weight control (U = 16462.00, *p* < 0.01). The remaining FCQ-SP dimensions were not significant regarding ON (*p* > 0.05).

In Table 4, the Spearman’s rho correlation was calculated to verify the relationship between the total scores on the ORTO-ES-11 and FCQ dimensions. A significant correlation was found for the total score on the ORTO-ES-11 and mood (*r* = 0.271, *p* < 0.01), the ORTO-ES-11 and natural content (*r* = 0.321, *p* < 0.01), the ORTO-ES-11 and weight control (*r* = 0.459, *p* <0.01) and ORTO-ES-11 and convenience (*r* = 0.118, *p* < 0.05).

## 4. Discussion

This study evaluated differences between individuals with a vegan, vegetarian, and omnivore diets with regard to ON and food choice motivations Our study revealed that 58.2% of vegans, 24.1% of vegetarians, and 17.7% of omnivores were at risk of ON. This suggests that individuals who have a restriction in their diet, such as in this case meat or animal products, are more likely to demonstrate a pathological preoccupation with healthy eating. However, we do not consider as a possible diagnostic criterion of ON the fact of reducing meat for compassionate or ethical reasons. In fact, the food restrictions associated with this pathology have another direction very different from the one defended by most philosophies in line with vegetarianism and go more in the direction of food purity. There is a continues debate between different researchers about the criteria of ON. The fact of studying individuals with different dietary restrictions can provide us with very valuable information even though the existence of a connection between ON and different types of diets is still not fully established. Recently, there has been an attempt to examine whether there is a possible cause–effect relationship between making specific dietary decisions and the appearance of ON [21,22]. A study performed by Brytek-Matera et al. [41] with seventy-nine individuals who followed a meatless diet and 41 omnivore individuals revealed that the individuals who followed a special diet (vegetarians and vegans) had a greater risk of suffering ON, which differs from those who did not follow a special diet. In contrast, another study developed by Cicekoblu and Tunçay [22] in a sample of 31 vegan/vegetarian individuals and 31 omnivore individuals concluded that the attitudes of individuals with dietary restrictions are not associated with a greater risk of suffering ON, as these types of diets are usually adopted based on ethical reasons, and not so much health-related. Barthel et al. [21] conducted another study performed on a sample of vegan individuals (*N* = 65) concluding that the vegan lifestyle was associated to ideas of animal wellbeing, politics and/or ecology, and not related the adoption of ON behavior. Our study finds that there were significantly more individuals on a vegan diet at risk of developing ON, although the instrument to detect the risk of ON is being widely criticized by the scientific community [14,17]. In this regard, not only diagnostic criteria are particularly important, but also the instruments established for their detection would play an essential role here.

An additional objective of the present study was to compare individuals with a vegan, vegetarian, and omnivore dietary patterns in terms of motives related to food choice. Our findings showed that individuals at risk of developing ON were less motivated in choosing foods based on their mood, health, and natural content and weight control than those presenting normal eating behaviors (no ON) and having no risk of ON. This would indicate that individuals at risk of developing ON use food to regulate their negative (e.g., stress) and positive (e.g., relaxation, feeling good) emotions and emotional states less than those having no risk of ON. Thus, it may be suggested that they regulate their emotions in a more adaptive way than individuals having no risk of ON. Confirmation of this hypothesis will require future studies. It is possible that in individuals having no risk of ON, food intake based on mood temporarily improves [42] (i.e., maintains emotional well-being through eating) and it may be related to emotional eating or external eating. Interestingly, contrary to our expectations, we found that lower levels of motivation for making food choices based on health and natural content were more prevalent in individuals at risk of developing ON than those presenting normal eating behaviors (no ON). This would suggest that individuals at risk of developing ON pay less attention to the prevention of chronic disease and general nutrition, and less concern with the use of additives and the selection of natural ingredients. These results are surprising considering that the main feature of ON is to promote and achieve health through the exclusion of entire food groups and strict avoidance of foods considered to be impure, unhealthy, or improper. The results may also suggest that motives related to food choice based on health and natural content measured by the FCQ-SP are associated with objectively health-related behaviors and beliefs. Individuals at risk of developing ON are less motivated in choosing foods based on weight control than those presenting normal eating behaviors (no ON). Weight control as an important motivational factor of food choice may be interpreted as indicating a greater concern for dietary restriction, skipping meals, or controlling food intake in order to maintain or lose weight. Our results suggested that concern about weight control did not play relevant role in ON.

Most authors agree that the reasons individuals select certain types of foods and, consequently, a specific diet could be key in the development of this pathology. Several factors can influence the decision to adopt a certain diet and, over the years, a growing population is now choosing to embrace such lifestyles. Thus, vegetarianism in its different facets is a clear global trend [16]. Some authors suggested that choosing a vegetarian diet for weight control could play a role in the etiology of an unbalanced diet [43]. Likewise, certain studies suggested that an appropriate vegetarian diet may be the most effective way of reducing body mass (expressed as BMI), improving the lipid profile in plasma, and decreasing the incidence of high blood pressure, cardiovascular disease, cerebrovascular accident, metabolic syndrome, and arteriosclerosis [44]. In contrast, a study [45] performed on 1320 adults was one of the few studies which displayed a negative relationship between vegetarianism and physical health, concluding that vegetarians may have greater probabilities of suffering cancer, allergies, a greater need for medical care and a worse quality of life.

Along these lines, our results revealed that, according to the Food Choice Questionnaire (FCQ-SP), several factors may be involved when selecting different types of foods, and thus defining an individual’s diet. Concretely, when comparing individuals with a risk of ON with those without such risk, independently of the selected diet, the significant dimensions were natural health and content, weight control and mood. In this sense, both natural health and content and weight control are dimensions which appear reflected, in one way or another, in the different studies that exist related to ON [46,47,48]. Bratman [8], who first described the term “orthorexia”, chose not to include weight control as a primary goal in the development of ON, but rather as a consequence of the selection of this type of diet. Following these lines, Barthels, Mayer and Pietrowsky [21] proposed that there may be weight-loss, however this does not have to be the main cause for reaching a diagnosis of ON, rather there are further symptoms and signs which may be associated with this disorder [21]. The fact that diet has to be pure and natural without any type of additives, conservatives and without meat products is one of the criteria proposed by Moroze et al. [13]. However, other researchers fail to comment on the absence of meat, focusing instead on the obsession with foods being free from impurities and healthy. From this viewpoint, vegetarians may consider meat products as being impure, thus also, the natural health content is significant when food choice is compared among the different types of diets. This would mean that these two dimensions somehow connect the risk of suffering ON vegetarianism or veganism, or rather, it should make us reflect upon the fact that ON continues to be the focus of researchers who attempt to clarify either the consequences or diagnostic criteria of this eating behaviour. Thus, the idea proposed by the researchers Depa, Barrada and Roncero [49] who advocate for distinguishing between healthy orthorexia and orthorexia nervosa could be a path for future research and should be considered when we have to face populations with specific diets. For example, in the case of vegetarians, where we cannot dismiss the existence of ON, but where it is important to clarify that a certain philosophy, in itself, or the adoption of a vegan diet are not risks, per se, with regards suffering pathological orthorexia; rather, these dietary habits could be framed within a healthy dimension of orthorexia.

This study has several limitations. First, we used a classification of vegetarianism and veganism based on self-reported eating behaviors. Therefore, our results may be specific for self-reported vegetarians and vegans and cannot be generalized to all those who follow a vegetarian or vegan diet. Self-reported vegetarians or vegans may, in fact, consume certain meat, fish and milk products. In addition, this was a cross-sectional analysis and, therefore, no causal inferences can be made, although it is possible to examine the coherence between data and the plausible causal models. Another possible weakness of this study is reflected by the limitations of the tool used to detect ON. Nonetheless, it is important to consider that this is the first study which evaluates ON behaviors among vegetarians, vegans, and omnivores in Spain. In addition, the recruitment of the participants was made by social networks where in terms of specific groups social media seemed to be more successful at recruiting, rather than a more general population. Finally, there is a lack of information on history of eating disorders or mental disorders diagnosis in our sample. Having recently been diagnosed or ever having been diagnosed with either eating disorders or other mental disorder could provide information whether individuals with ON have a co-occurring condition.

Further studies are required to determine whether different types of diets with food restrictions can entail ON behaviors. A broader knowledge on ON may help determine the potential lines of action for the prevention of this disorder among different populations, considering and respecting a person’s ideas and ethical principles, when referring to the choice to include or abstain from the consumption of certain types of foods.

## 5. Conclusions

Our results showed that there was a link between the adoption of different dietary patterns and ON behaviors. More individuals following a vegan diet (58.2%) were at risk of developing ON. In addition, the attitudes of vegans and vegetarians towards eating were associated with ON behaviors and the main motivation for adopting this type of diet was the natural and healthy content of the food consumed. Further research is necessary to expand our findings using a larger population and with more reliable tools (such as the Düsseldorf Orthorexia Scale or the Eating Habits Questionnaire) in order to expand our knowledge about this pathological preoccupation with healthy eating.

More information on dietary patterns could help set appropriate dietary standards and guidelines both individuals following a vegetarian/vegan diet and individuals following an omnivorous diet. Some food choice motives (e.g., mood, weight control) can have negative effects while other motives can have beneficial effects. The consideration of individual motives and food choices provides important for benchmarks not only for evaluating overall diet quality [50] but also for providing effective treatment to individuals with different dietary patterns.

## Figures and Tables

**Table 1:** Sociodemographic characteristics of the sample and relationships with type of diet by the Chi-squared test.

Variables	Vegans		Vegetarians		Omnivores		X^2^ (*p* Value)
	*N*	%	*N*	%	*N*	%	
Female	79	78.2	90	82.6	185	72.3	4.80 (0.09)
Male	22	21.8	19	17.4	71	27.7	
Single	81	80.2	96	88.1	167	65.2	23.48 (0.00)
Married	20	19.8	13	11.9	89	34.8	
BMI lower than 18.5 kg/m^2^ (underweight range)	11	11.2	6	5.6	12	4.8	15.05 (0.02)
BMI of 18.5 kg/m^2^–24.99 kg/m^2^ (normal range)	68	69.4	78	72.2	153	60.7	
BMI upper than 25 kg/m^2^–29.9 kg/m^2^ (overweight range)	12	12.2	18	16.7	65	25.8	
BMI upper than 30 kg/m^2^ (obese range)	7	7.1	6	5.6	22	8.7	
Non risk of Orthorexia (having a score > 25)	44	19.2	67	29.3	118	51.5	8.35 (0.01)
Risk of ON (having a score < 25)	138	58.2	57	24.1	42	17.7	

**Table 2:** The distribution of the Food Choice Questionnaire (FCQ-SP) Spanish version scores of the vegans, vegetarians, and omnivores according to Kruskal–Wallis H test.

Dimensions	Type of Diet	*N*	M (SD) ^1^	(Min-Max)	Sum of Ranks	*H*	*p*
Price	Vegan	101	15.8 (3.3)	(3.21)	252.66	4.63	0.098
	Vegetarian	109	15.6 (3.2)	(4.21)	243.40		
	Omnivorous	256	15.0 (3.6)	(3.21)	221.73		
Mood	Vegan	101	27.4 (7.0)	(6.42)	218.47	1.19	0.123
	Vegetarian	109	27.4 (6.9)	(6.41)	220.31		
	Omnivorous	256	28.5 (7.0)	(6.42)	245.04		
Health and natural content	Vegan	101	49.5 (7.1)	(26.62)	263.71	8.76	0.012
	Vegetarian	109	47.8 (8.9)	(12.62)	241.40		
	Omnivorous	256	46.3 (9.1)	(9.63)	218.22		
Sensory appeal	Vegan	101	22.0 (3.7)	(11.28)	218.87	11.37	0.003
	Vegetarian	109	21.6 (3.9)	(7.28)	203.82		
	Omnivorous	256	22.8 (3.5)	(4.28)	251.91		
Weight control	Vegan	101	11.2 (4.7)	(3.21)	172.66	40.38	0.000
	Vegetarian	109	12.8 (3.8)	(3.21)	210.17		
	Omnivorous	256	14.3 (4.0)	(3.21)	267.44		
Convenience	Vegan	101	24.7 (5.0)	(14.35)	230.50	1.24	0.536
	Vegetarian	109	25.2 (5.4)	(7.35)	229.34		
	Omnivorous	256	24.5 (5.6)	(5.35)	229.34		
Familiarity	Vegan	101	15.3 (4.4)	(5.26)	207.47	37.28	0.000
	Vegetarian	109	14.2 (4.8)	(4.26)	179.30		
	Omnivorous	256	17.3 (4.8)	(4.27)	266.85		

^1^ df = (2465); Min = Minimum, Max = Maximum.

**Table 3:** The distribution of FCQ-SP scores according to the results of Mann–Whitney U test for the risk of orthorexia and lack of ON (no ON).

Dimensions	Orthorexia	*N*	M (SD)	(Min–Max)	Sum of Ranks	U	*p*
Price	Risk of Orthorexia	237	15.2 (3.5)	(3.21)	54,596.50	26,393.50	0.607
	No Orthorexia	229	15.4 (3.4)	(3.21)	54,214.50		
Mood	Risk of Orthorexia	237	26.8 (7.1)	(6.42)	49,568.00	21,365.00	0.000
	No Orthorexia	229	29.3 (6.7)	(6.42)	59,243.00		
Natural content	Risk of Orthorexia	237	45.2 (8.9)	(9.63)	47,310.50	19,107.50	0.000
	No Orthorexia	229	49.5 (8.0)	(12.63)	61,500.50		
Sensory appeal	Risk of Orthorexia	237	22.3 (3.8)	(7.28)	55,613.00	26,863.00	0.850
	No Orthorexia	229	22.4 (3.6)	(4.28)	53,198.00		
Weight control	Risk of Orthorexia	237	11.9 (4.2)	(3.19)	44,665.00	16,462.00	0.000
	No Orthorexia	229	14.7 (4.0)	(3.21)	64,146.00		
Convenience	Risk of Orthorexia	237	24.5 (5.5)	(5.35)	53,568.50	25,455.50	0.247
	No Orthorexia	229	24.9 (5.4)	(6.35)	55,152.50		
Familiarity	Risk of Orthorexia	237	16.1 (4.8)	(4.27)	55,334.00	27,131.00	0.997
	No Orthorexia	229	16.2 (5.08)	(4.27)	53,477.00		

Min = Minimum, Max = Maximum.

**Table 4:** Spearman’s correlations between the Spanish version of the ORTO-15 (ORTO-ES-11) and FCQ-SP dimensions.

	1	2	3	4	5	6	7	8
1.- Orthorexia								
2.- Price	0.048							
3.- Mood	0.271 **	0.267 **						
4.- Natural content	0.321 **	0.134 **	0.450 **					
5.- Sensory appeal	0.016	0.217 **	0.376 **	0.244 **				
6.- Weight control	0.459 **	0.162 **	0.415 **	0.399 **	0.195 **			
7.- Convenience	0.118 *	0.520 **	0.329 **	0.113 *	0.212 **	0.237 **		
8.- Familiarity	0.041	0.112 *	0.492 **	0.410 **	0.332 **	0.329 **	0.145 **	

* *p* < 0.05; ** *p* < 0.01.
